# A Call for Unity in the Neuroscience Community

**DOI:** 10.1523/ENEURO.0148-25.2025

**Published:** 2025-04-25

**Authors:** Christophe Bernard

**Affiliations:** Editor in Chief, *eNeuro*

The scientific landscape in the United States is experiencing a significant shift. Recent developments have created new challenges for US researchers, US institutions, and scientific societies worldwide that warrant our collective attention and thoughtful response. These changes present an opportunity to reaffirm the fundamental importance of scientific exchange.

## The Current Landscape for Neuroscience in the United States

Recent policy changes have significantly altered funding for biomedical research in the United States. The National Institutes of Health (NIH) has announced substantial reductions in funding and has canceled study sections. Although the story is still unravelling, the decision to limit the overheads to 15% may threaten the very existence of some laboratories with obvious disastrous human consequences. This also includes the closure of NIH-core funded facilities and the firing of scientific personnel.

## Impact on International Collaboration

The current climate has changed the way international researchers consider future travel to the United States. Being based in Europe, I have observed a reluctance among some scientists to participate in US conferences. In a recent incident, a CNRS researcher was denied entry to the United States because of conversations on his phone (French Researcher, 2025). French researchers received a notification to be extra careful if they are planning to go the United States, including a recommendation not to take one's phone and computer or to erase any “sensitive” information. This kind of recommendation from the French Department of Defense and Security may act as a strong deterrent to travel to the United States. Other EU countries and China have issued similar warnings about travel to the United States (Croucher and Rahman, 2025; Laws, 2025), in essence recommending travelers to comply strictly with entry rules given the risk of detention or deportation. Conversely, US-based scientists may be prevented from attending meetings outside the United States.

Neuroscience has historically benefited from diverse international perspectives and expertise, and I am concerned that these obstacles to international travel will damage that collaboration.

## The Role of Scientific Societies in Providing Support in Troubled Times

Scientific societies can act as stabilizers as they are the natural conduit for knowledge dissemination, professional development, and community building that transcends political cycles and policy fluctuations. For example, the annual meeting of the Society for Neuroscience represents more than just a gathering—it constitutes an important forum for sharing and exchanging. It is considered by many neuroscientists worldwide as the flagship neuroscience meeting. Many such meetings occur every year in all neuroscience subfields and of course in all fields of science. Boycotting the meetings we usually attend would serve no purpose. We would punish ourselves doing it; I do not think that the present US government cares whether or not non-US-based scientists attend a scientific meeting.

## A Collective Responsibility to Support Neuroscience

The current situation presents not only challenges but also an opportunity to demonstrate the resilience and solidarity of the neuroscientific community. Supporting US-based societies and meetings during troubled times for our US colleagues represents an investment to maintain a structure we all need. For both US- and non-US-based scientists, this means continuing to prioritize participation in scientific meetings and society activities even when budgets are constrained. We all should adopt a proactive approach to building resilience rather than merely (over)reacting to the present situation, as bad as it may be.

Boycotting scientific meetings would achieve the opposite to what the community needs. It would jeopardize non-profit institutions without any benefit. This moment calls for unity rather than division, for constructive engagement rather than disengagement. Whatever one's political perspectives, the advancement of neuroscience research serves our collective interests. By standing together to support scientific societies, we affirm our commitment to these shared goals. If this means being extra careful by strictly complying with US entry rules, it is a small price to pay to show our support to our US colleagues. On a personal note, I plan to attend my two most important yearly meetings, the annual meetings of the Society for Neuroscience and of the American Epilepsy Society. I hope to see you all there.
